# Efficiency of Electronic Health Record Assessment of Patient-Reported Outcomes After Cancer Immunotherapy: A Randomized Clinical Trial

**DOI:** 10.1001/jamanetworkopen.2022.4427

**Published:** 2022-03-31

**Authors:** Liyan Zhang, Xiaotian Zhang, Lin Shen, Dan Zhu, Saili Ma, Lin Cong

**Affiliations:** 1 Key Laboratory of Carcinogenesis and Translational Research (Ministry of Education), Department of GI Medical Oncology, Peking University School of Oncology, Beijing Cancer Hospital & Institute, Beijing, China; 2 Aistarfish Technology Co, Ltd, Zhejiang, China

## Abstract

**Importance:**

Cancer immunotherapy causes a wide range of immune-related adverse events (irAEs) that require close and timely follow-up.

**Objectives:**

To compare the efficiency between electronic patient-reported outcomes (ePRO) and traditional follow-up models in cancer immunotherapy.

**Design, Setting, and Participants:**

This open-label randomized clinical trial was performed from September 1, 2019, to March 31, 2021. Patients were randomized to the ePRO model intervention or a control group by a computer system. A total of 28 Chinese tertiary care hospitals participated. Patients who were receiving cancer immunotherapy and could use smartphones or computers were eligible. A total of 300 patients were screened and 278 (92.7%) were enrolled.

**Interventions:**

The control group was followed up using traditional methods, including clinic visits every 21 days and telephone follow-up every 3 months. In the intervention group, the ePRO follow-up model included a questionnaire of common symptoms and an image recognition function to evaluate grades of typical irAEs. Patients completed questionnaires weekly and uploaded pictures of results between visits. When grade 1 or 2 irAEs occurred, standardized advice was sent automatically. If grade 3 or 4 irAEs were reported, the model alerted the health care team for assessment and intervention immediately. All patients were followed up for 6 months or until treatment completion.

**Main Outcomes and Measures:**

Incidence of serious (grades 3 to 4) irAEs, emergency department (ED) visits, quality of life (QOL), time spent implementing the ePRO model, rate of treatment discontinuation, and death were compared between groups post intervention.

**Results:**

A total of 278 patients (mean [SD] age, 58.8 [12.7 (range, 27-78)] years; 206 men [74.1%]) were included in the analysis, consisting of 141 in the intervention group and 137 in the control group. At the postintervention evaluation, the intervention group showed a reduced incidence of serious irAEs (29 of 141 [20.6%] vs 46 of 137 [33.6%]; hazard ratio [HR], 0.51 [95% CI, 0.30-0.88]; *P* = .01), fewer ED visits (23 of 141 [16.3%] vs 41 of 137 [29.9%]; HR, 0.46 [95% CI, 0.26-0.81]; *P* = .01), a lower rate of treatment discontinuation (5 of 141 [3.6%] vs 15 of 137 [11.0%]; HR, 0.30 [95% CI, 0.11-0.85]; *P* = .02), a higher QOL level (mean [SD] score, 74.2 [15.1; 95% CI, 71.7-76.9] vs 64.7 [28.5; 95% CI, 61.0-68.4]; *P* = .001), and less time implementing follow-up (mean [SD], 8.2 [3.9; 95% CI, 5.0-10.6] minutes vs 36.1 [15.3; 95% CI, 33.6-38.8] minutes; *P* < .001). However, there were no significant differences between groups in death rates (2 of 141 [1.4%] vs 5 of 137 [3.6%]; HR, 0.38 [95% CI, 0.07-1.99]; *P* = .28).

**Conclusions and Relevance:**

This randomized clinical trial found that the ePRO follow-up model can improve safety and QOL of patients receiving cancer immunotherapy as well as reduce time spent monitoring. This model may provide reliable information and management recommendations.

**Trial Registration:**

Chinese Clinical Trial Registry Identifier: ChiCTR2100052819

## Key Points

**Question:**

Can an electronic patient-reported outcome (ePRO) model improve outcome in cancer immunotherapy compared with a traditional model?

**Findings:**

In this randomized clinical trial of 278 patients from 28 Chinese hospitals comparing ePRO and traditional follow-up models, the intervention group showed a reduced incidence of serious immune-related adverse events, fewer emergency department visits, a lower rate of treatment discontinuation, a higher quality of life, and less time needed to implement the model.

**Meaning:**

These findings suggest that the ePRO follow-up model is efficient and may provide reliable information and management recommendations in patients receiving immunotherapy for cancer.

## Teaser

This randomized clinical trial investigates the efficiency of an application for an electronic patient-reported outcome follow-up system compared with a traditional model for safety and quality of life among patients receiving cancer immunotherapy.

## Introduction

### Cancer Immunotherapy and Immune-Related Adverse Events

The rapid advances in cancer immunotherapy using immune checkpoint inhibitors (ICIs) have led to significantly improved survival of patients.^1,2^ At the same time, ICIs have been associated with multiple immune-related adverse events (irAEs).^3^ The irAEs can affect a wide range of organs and induce nonspecific symptoms with delayed onset^4^ and prolonged duration that are easily neglected, which may lead to life-threatening disorders. The incidence of irAEs of any grade attributable to single-agent ICI therapy is as high as 90%.^5,6^ Among these, irAEs of grade 3 or higher occurred in approximately 20% to 43% of patients,^7^ and 2% of the patients died due to irAEs worldwide.^8^ Hence, patients and clinicians should be well informed and vigilant to ensure that irAEs are recognized and interventions are performed in a timely manner.

### Follow-up of Patients Experiencing irAEs

Immune-related adverse events are more likely to be experienced outside health care settings and therefore need to be followed up effectively. Because of the large amount of data generated and the long period of time needed, an efficacious, less labor-intensive, time-saving follow-up model is needed. However, the number of studies, especially large-scale randomized clinical trials, are limited. Hoffner and Rubin^9^ reported improvements in telephone triage by using immuno-oncology–essential materials^10^ to reduce the burden of irAE follow-up. Le et al^11^ evaluated the impact of a pharmacist-led irAE follow-up process that reduced the times required for physicians to manage irAEs and increased physician confidence.

As a new follow-up model, electronic patient-reported outcomes (ePROs), including health-related questionnaires completed by patients, could effectively capture symptoms and their severity. Studies have shown that the ePRO model enables timely, cost-effective, and continuous collection of patient symptom data.^12,13,14,15^ Furthermore, Basch et al^16^ found that the ePRO model used for management of patients with cancer could result in improved quality of life (QOL), fewer emergency department (ED) visits, longer duration of palliative chemotherapy, and superior quality-adjusted survival. However, only 1 study^17^ assessed an eHealth intervention system used for 50 patients with malignant melanomas receiving cancer immunotherapy. The study reported that the level of satisfaction with eHealth intervention was high among patients and clinicians.

However, the follow-up efficiency of an ePRO model for improving safety for patients receiving cancer immunotherapy is currently under debate, especially for a lack of evidence derived from large sample populations or randomized clinical trials. This study aimed to investigate the efficiency of the ePRO follow-up system for improving the safety and QOL of patients receiving cancer immunotherapy and making the follow-up process less time consuming.

## Methods

### Trial Design

This study was an open-label, multicenter randomized clinical trial comparing the outcomes of intervention and control group patients who were treated with cancer immunotherapy. A complete copy of the trial protocol is provided in Supplement 1. We prospectively included patients who received cancer immunotherapy from September 1, 2019, to March 31, 2021, in 28 tertiary care hospitals in 15 provinces in China. Patients were from southern, eastern, and northern China. Patients were randomized into the intervention and control groups by a computer system. This study was approved by the Beijing Cancer Hospital institutional review board. All participants provided written informed consent. This study followed the Consolidated Standards of Reporting Trials (CONSORT) reporting guideline.

### Participants

Enrollment ended in March 2021 when the last patients had their 6-month follow-up. Eligible individuals for the study were 18 years or older and were receiving cancer immunotherapy; had an Eastern Cooperative Oncology Group performance status of 0 or 1 and life expectancy of at least 6 months; were willing to complete the follow-up process according to the protocol; and could use smartphones or computers to include information in the model application (app) with or without the help of their caregivers. Exclusion criteria consisted of an uncontrolled medical disorder (hepatic failure, kidney failure, respiratory failure, or heart failure), diagnosed mental disease, and refusal to participate.

### Interventions

#### Follow-up Team

The ePRO app was built and maintained by 2 computer specialists from the AI Startfish Technology Co, Ltd (D.Z. and S.M.) (eMethods 1 in Supplement 2). A follow-up team that included an oncology specialist and 2 nurses was established in every center. Every center had a unique account for the ePRO app, and each team was responsible for follow-up to their own patients. All researchers had at least 2 years of experience in ICI therapy and received training about the research program, use of the app, and the follow-up process before the study commenced.

#### Control Group

Researchers educated patients and their caregivers about immunotherapy and common symptoms of irAEs at baseline. Patients were followed up using a traditional model, including clinic visits every 21 days and via the telephone every 3 months. Patients could visit the clinic when they felt any discomfort.

#### Intervention Group

Patients in the intervention group were registered and followed up via the ePRO app. The app contained a questionnaire of common symptoms and an image recognition function of examination results to evaluate the occurrence and grades of typical irAEs according to the guidelines.^3,17,18,19,20^ Patients checked the questionnaire weekly and uploaded pictures of examination results between visits on their mobile device or computer. The app sent 3 reminder messages on 3 consecutive days to patients who did not reply. If no reply was obtained, follow-up by telephone was conducted.

**Figure 1.**
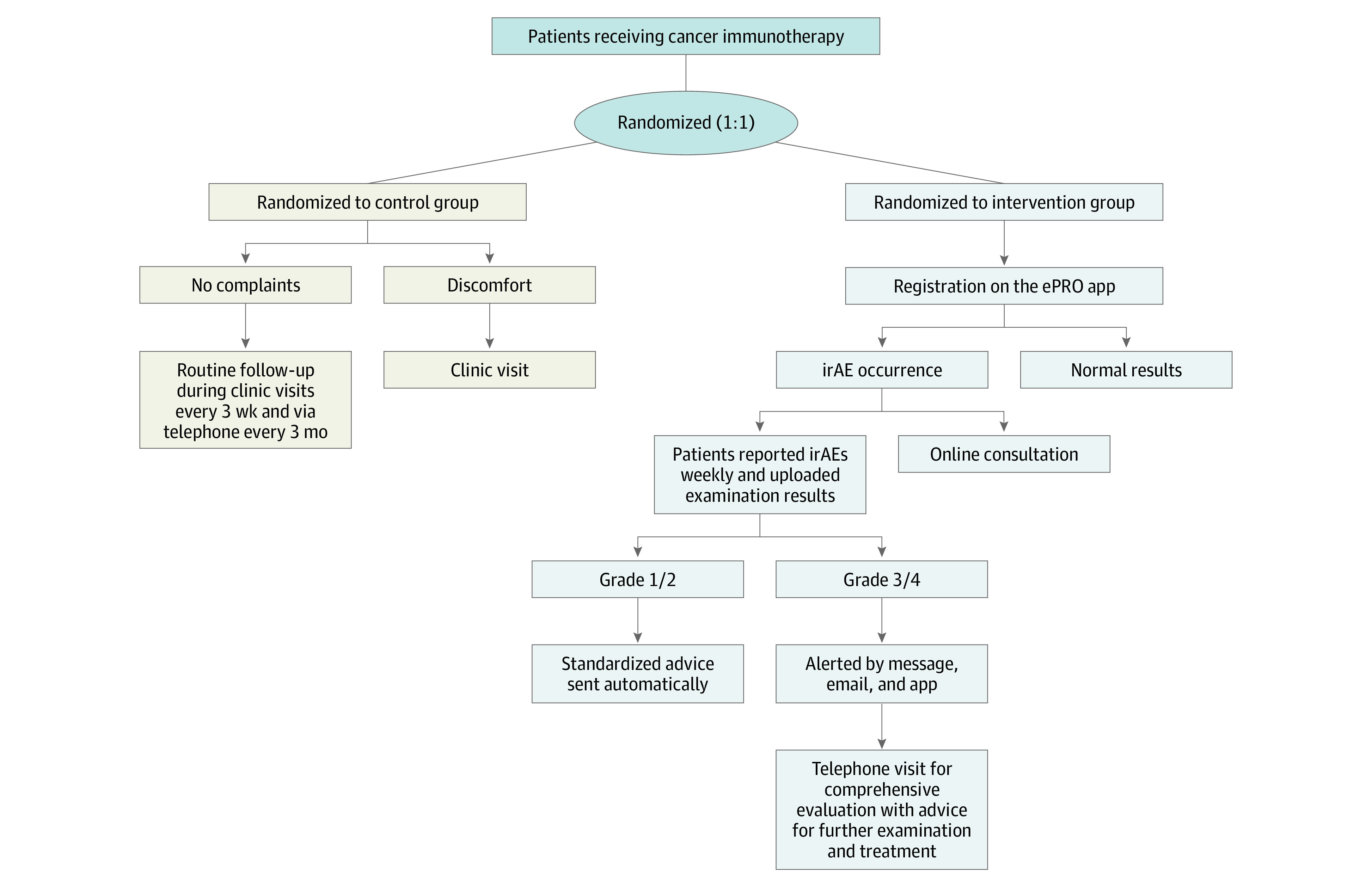
Study Route The control group received the traditional model of follow-up; the intervention group received the electronic patient-reported outcome (ePRO) model, follow-up monitoring, and consultation via the ePRO application (app). irAE indicates immune-related adverse effect; serious irAEs include grades 3 to 4.

The app also had an algorithm for assessing severity of symptoms or examination results according to National Cancer Institute–Common Terminology Criteria for Adverse Events, version 5.0.^3,18,19,20^ (eMethods 2 in Supplement 2). When grade 1 or 2 irAEs occurred, the standardized advice from the irAE management guidelines^3,17,18,19,20^ was sent to patients via the app automatically. If grade 3 or 4 irAEs were reported, the app alerted the health care team via text message, email, and app simultaneously. The health care team followed up via telephone and comprehensively evaluated the patient’s situation. If serious irAEs were still suspected, patients were advised to go to the hospital or ED for evaluation and treatment. In addition, patients could consult the team on the app anytime (Figure 1).

### Outcomes and Measures

#### Relative Indexes of irAEs

We collected data regarding irAE incidence and grades, the rate of occurrence of grade 3 or 4 irAEs (number of cases, with the irAEs divided by the total number of cases), ED visits, and rate of treatment discontinuation and death owing to irAEs. These data were compared between the intervention and control groups.

#### Quality of Life

Quality of life was assessed using the European Organization for Research and Treatment of Cancer QLQ-C30 validated by Aaronson et al^21^ and used to measure the QOL of patients every 3 months. The QLQ-C30 was translated into Chinese by Wan et al^22^ and consisted of 30 items, including 5 functional dimensions (physical, role, emotional, perception, and social functions), 9 symptom dimensions, and 2 health situation dimensions. The 2 health situation items were assigned scores ranging from 1 to 7, whereas the other items were evaluated using a 4-grade Likert score. Based on the rules for assigning scores, we transformed every dimensional score to a standard score ranging from 0 to 100. The functional dimension score and the health situation score were positively correlated with patient QOL.

#### Mean Time per Follow-up Session

Researchers recorded the time spent on each follow-up session. The mean time spent on follow-up (total time divided by the number of follow-up sessions per patient) was compared between the intervention and control groups.

### Sample Size

We used PASS software, version 10.0 (NCSS Statistical Software), to calculate the sample size. It was assumed that the incidence of severe adverse reactions in the intervention group would be 10% lower than that in the control group. The unilateral *z* test was applied; α = .05 and β = 0.1. The results showed that 103 patients were needed in each group; considering loss to follow-up, the total sample included 300 cases.

### Randomization

Randomization occurred immediately after a participant provided written informed consent at each center. Participants were randomly assigned to the control or intervention group using Interactive Response Technology software, version 3.0 (Almac Clinical Technologies). Each patient’s group randomization was based on a site-specific, sequentially numbered study identification number at enrollment. Randomization was stratified based on study site to address potential center effect or bias. This protocol helped to mitigate biases that might stem from differences in patient populations, care management, and other contextual factors that may be unique to an individual study site.

### Blinding

Patients were not blinded to their group assignment. To mitigate potential bias caused by nonblinding, we chose objective indicators such as irAEs and the QOL questionnaire as the primary outcome measures for their excellent interrater reliability, external validity, and minimal vulnerability to rater bias.^21,22^

### Statistical Analysis

All data were input into SPSS Statistics software, version 20.0 (IBM Corp), by 2 researchers (L.Z. and D.Z.). Descriptive statistics were used for the demographic characteristics, clinical conditions, irAE relative indexes, and QOL. Enumeration data were expressed as frequency and percentage, whereas quantitative data were expressed as mean (SD). Variance independent-sample *t* test and χ^2^ test were used to compare the differences in enumeration and quantitative data, respectively, between groups. Two-sided *P* < .05 indicated statistical significance.

## Results

### Recruitment

**Figure 2.**
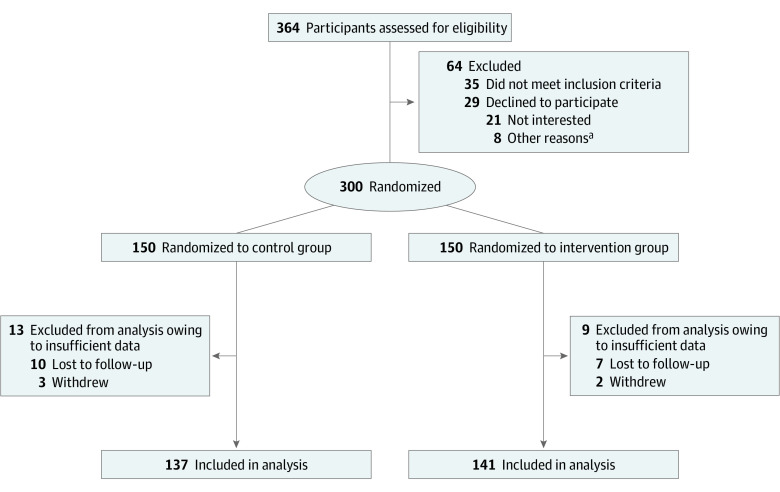
Participant Recruitment Flow Diagram ^a^Other reasons include time commitment, poor memory, and poor skills with use of computers and electronic devices.

A total of 364 patients were screened for eligibility; of these, 35 did not meet the inclusion criteria and 29 declined to participate. The remaining 300 eligible patients agreed to participate. The participant flow diagram is presented in Figure 2. Among these, 150 patients were randomized to the control group and 150 to the intervention group. Data from 9 patients from the intervention group and 13 from the control group were excluded from the analysis owing to insufficient data. Consequently, data from 278 patients (92.7%), including 141 in the intervention group and 137 in the control group, were included in the final analysis (206 men [74.1%] and 72 women [25.9%]; mean [SD] age, 58.8 [12.7 (range, 27-78)] years) (Figure 2).

### Baseline Characteristics

Among the 278 patients, 269 (96.8%) were married, and 126 (45.3%) had attained a college degree or greater level of education. The 3 most common cancer types were gastric (87 [31.3%]), esophageal (60 [21.6%]), and lung (32 [11.5%]). In addition, 183 (65.8%) patients received immune monotherapy; 45 (16.2%), a combination of target agents; 42 (15.1%), a combination of chemotherapeutic agents; and 8 (2.9%), a combination of other immune agents. Overall, baseline data were well balanced between study groups (Table 1).

**Table 1.** Baseline Patient Characteristics

Characteristic	Patient group^a^	
	ePRO intervention (n = 141)	Control (n = 137)
Age, mean (SD), y	57.6 (12.6)	60.1 (12.7)
Sex		
Men	106 (75.2)	100 (73.0)
Women	35 (24.8)	37 (27.0)
Marital status		
Married	138 (97.9)	131 (95.6)
Single, divorced, or other	3 (2.1)	6 (4.4)
Educational level		
Junior middle school or below	23 (16.3)	29 (21.2)
High middle school	50 (35.5)	50 (36.5)
College or above	68 (48.2)	58 (42.3)
Cancer site		
Gastric	49 (34.7)	38 (27.7)
Esophageal	34 (24.1)	26 (19.0)
Lung	13 (9.2)	19 (13.9)
Pancreatic	11 (7.8)	15 (11.0)
Colorectal	7 (5.0)	10 (7.3)
Breast	6 (4.3)	13 (9.5)
Brain	6 (4.3)	3 (2.2)
Liver	4 (2.8)	4 (2.9)
Kidney	3 (2.1)	0
Other	8 (5.7)	9 (6.6)
Treatment method		
Immune monotherapy	95 (67.4)	88 (64.2)
Combined target agents	22 (15.6)	23 (16.8)
Combined chemotherapy	20 (14.2)	22 (16.1)
Combined other immune agents	4 (2.8)	4 (2.9)

Abbreviation: ePRO, electronic patient-reported outcome.

^a^ Unless otherwise indicated, data are expressed as number (%) of patients. Percentages are rounded and may not total 100.

### Outcomes and Estimation

#### Relative Indexes of irAEs

The overall incidence of irAEs was 228 of 278 patients (82.0%). The most frequent irAEs were dermatologic (77 [27.7%]), pneumonitis (50 [18.0%]), and musculoskeletal (34 [12.2%]). Moreover, 64 patients (23.0%) visited the ED and 7 (2.5%) died of severe irAEs. Compared with the control group, the intervention group had fewer severe irAEs (29 of 141 [20.6%] vs 46 of 137 [33.6%]; hazard ratio [HR], 0.51 [95% CI, 0.30-0.88]; *P* = .01), fewer ED visits (23 of 141 [16.3%] vs 41 of 137 [29.9%]; HR, 0.46 [95% CI, 0.26-0.81]; *P* = .01), and less treatment discontinuation due to irAEs (5 of 141 [3.6%] vs 15 of 137 [11.0%]; HR, 0.30 [95% CI, 0.11-0.85]; *P* = .02). The death rate was lower in the intervention group, but the difference was not significant (2 of 141 [1.4%] vs 5 of 137 [3.6%]; HR, 0.38 [95% CI, 0.07-1.99]; *P* = .28), as shown in Table 2.

**Table 2.** irAE Relative Indexes After Intervention

irAE index	Patient group, No. (%)^a^		χ^2^ value	HR (95% CI)	*P* value
	ePRO intervention (n = 141)	Control (n = 137)			
IrAE occurrence					
Yes	111 (78.7)	117 (85.4)	2.101	0.63 (0.34-1.18)	.16
No	30 (21.3)	20 (14.6)			
Severe irAE					
Yes	29 (20.6)	46 (33.6)	5.969	0.51 (0.30-0.88)	.01
No	112 (79.4)	91 (66.4)			
ED visits					
Yes	23 (16.3)	41 (29.9)	7.268	0.46 (0.26-0.81)	.01
No	118 (83.7)	96 (70.1)			
Treatment discontinuation					
Yes	5 (3.6)	15 (11.0)	5.703	0.30 (0.11-0.85)	.02
No	136 (96.5)	122 (89.1)			
Death					
Yes	2 (1.4)	5 (3.6)	1.409	0.38 (0.07-1.99)	.28
No	139 (98.6)	132 (96.3)			

Abbreviations: ED, emergency department; ePRO, electronic patient-reported outcome; HR, hazard ratio; irAE, immune-related adverse event.

^a^ Percentages have been rounded and may not total 100.

#### Quality of Life

There were no significant differences in QOL scores at baseline and 3 months. However, total mean (SD) scores were higher for the intervention group at 6 months (74.2 [15.1 (95% CI, 71.7-76.9)] vs 64.7 [28.5 (95% CI, 61.0-68.4)]; *P* = .001), especially for the physical (84.9 [10.5 (95% CI, 82.9-88.5)] vs 68.8 [20.7 (95% CI, 65.8-72.5)]; *P* < .001) and emotional (81.0 [12.8 (95% CI, 76.3-84.6)] vs 69.9 [28.3 (95% CI, 63.5-73.5)]; *P* = .04) dimensions (Table 3).

**Table 3.** QOL Between Groups Before and After Intervention

Dimension	QOL score, mean (SD) [95% CI]^a^						*P* value between-group comparison			
	ePRO intervention group (n = 141)			Control group (n = 137)			All times	Baseline	3 mo	6 mo
	Baseline	3 mo	6 mo	Baseline	3 mo	6 mo				
Total situation	61.3 (12.7) [66.5-73.3]	66.2 (10.2) [66.1-71.4]	74.2 (15.1) [71.7-76.9]	60.7 (15.8) [56.0-71.9]	61.7 (12.1) [60.3-72.8]	64.7 (28.5) [61.0-68.4]	.008	.87	.82	.001
Physical function	69.6 (8.26) [65.6-72.4]	72.2 (16.0) [65.6-75.5]	84.9 (10.5) [82.9-88.5]	70.6 (13.4) [65.5-72.5]	69.1 (11.2) [65.4-72.6]	68.8 (20.7) [65.8-72.5]	<.001	.87	.90	<.001
Role function	68.7 (18.1) [64.6-74.8]	68.0 (20.1) [65.1-72.3]	70.9 (13.4) [67.3-73.2]	69.1 (21.3) [66.5-75.8]	67.0 (14.1) [64.8-70.5]	69.9 (32.4) [67.9-73.2]	.23	.49	.48	.13
Social function	66.8 (11.3) [63.8-73.5]	68.6 (13.2) [62.5-74.8]	69.7 (15.8) [64.1-72.1]	67.9 (11.3) [62.9-69.8]	68.4 (11.3) [65.5-75.5]	69.1 (10.3) [64.2-73.6]	.15	.41	.56	.81
Emotional function	69.6 (18.2) [66.3-75.8]	73.4 (12.1) [67.5-77.6]	81.0 (12.8) [76.3-84.6]	70.4 (19.4) [64.3-76.5]	69.8 (17.3) [64.5-73.3]	69.9 (28.3) [63.5-73.5]	<.001	.58	.62	.04
Perception function	70.3 (13.0) [68.5-75.1]	69.7 (18.4) [65.3-74.8]	72.0 (7.84) [67.8-77.6]	69.9 (14.0) [64.8-73.3]	69.4 (15.9) [66.9-74.4]	70.5 (24.1) [67.2-73.1]	.41	.55	.49	.17

Abbreviations: ePRO, electronic patient-reported outcome; QOL, quality of life.

^a^ Scores range from 0 to 100, with higher scores indicating better QOL.

#### Time Expended

During the entire follow-up process, the mean time expended for each follow-up for the intervention and control groups ranged from 0 to 120 and 10 to 100 minutes, respectively. The mean (SD) times were 8.2 (3.9 [95% CI, 5.0-10.6]) minutes vs 36.1 (15.3 [95% CI, 33.6-38.8] minutes; *P* < .001). Thus, the mean time expended in the intervention group was significantly shorter.

## Discussion

The optimal treatment of patients receiving ICIs could be achieved via the comprehensive assessment, grading, and long-term surveillance of symptoms. An efficient follow-up process that facilitates the monitoring and reporting of irAEs in a timely manner is important for ensuring patient safety. Previous studies have shown that ePRO follow-up enables timely and continuous collection of information regarding symptoms in a cost-effective manner^9,10,11,12,13,14^ and results in an improved QOL, fewer ED visits, longer duration of palliative chemotherapy, and superior quality-adjusted survival.^15,16^ However, the efficacy of ePRO follow-up has not been examined in patients treated with ICIs. The purpose of this study was to investigate the efficiency of the ePRO model in a randomized clinical trial in a mixed sample from multiple centers.

### Safety

Our findings show that patients in the intervention group had a trend of fewer severe irAEs, fewer ED visits, less treatment discontinuation, and lower death rates, although no statistically significant differences were observed. Because of the nonspecific performance and delayed onset, detection of irAEs was crucial for ensuring patient safety. Compared with routine follow-up, weekly ePRO follow-up was performed more frequently. It can facilitate an early response to patient symptoms, thereby preventing occurrence of severe irAEs. In addition, severe irAEs are also nonspecific irrespective of their level of lethality, and the ePRO follow-up system can recognize signs of irAEs and alert medical personnel immediately after a patient provides a report. Patients are then contacted and alerted in a timely manner, which makes it possible for a patient to undergo treatment and avoid fatal consequences. Moreover, patients should discontinue immunotherapy immediately after the occurrence of severe irAEs. Early response to mild irAEs can prevent the aggravation of symptoms and the occurrence of severe irAEs. Hence, a decrease in the rate of treatment discontinuation might help improve disease progress.

### Patient QOL

At 6 months after the intervention, the overall QOL, physical functions, and emotional functions of patients in the intervention group were higher than those in the control group. These findings were similar to those of a previous study.^16^ One of the possible reasons included the periodic and timely follow-up process that may help to reduce the occurrence of irAEs and increase the advice of safe care for mild irAEs that may alleviate patient symptoms, thus improving physical function. In addition, medical team responses (via standardized advice, telephone, or online consultation) to patients’ symptoms in a timely manner may have helped to relieve patient anxiety, depression, or other negative emotional issues caused by a lack of knowledge, thereby improving overall patient QOL.

### Time Expended

To further explore the efficiency of the ePRO model, we also compared the time necessary to use it and the routine follow-up model, which has not been compared in previous studies. The results show that the ePRO model was less time consuming than the traditional follow-up process. One possible reason for this difference could be that the ePRO follow-up model, which is based on the information system, mainly uses the platform to automatically send follow-up tasks and standardized advice. Only a small group of patients who did not respond or were suspected to have serious irAEs were followed up via telephone. Compared with the traditional telephonic follow-up process for each patient, the ePRO approach undoubtedly saves time and labor required for recording, planning, and reviewing the follow-up arrangements for patients, especially with mobile phone connectivity issues, language barriers, hearing loss issues, and other communication-related difficulties.

In addition, the system can simultaneously perform automatic recognition of symptoms, assessment of examination results, and timely warnings, thereby reducing the time required by medical staff to check the test results. Given the large clinical workload and shortage of nursing staff, the ePRO follow-up model can effectively reduce follow-up time and improve work efficiency.

### Limitations

Our study has some limitations. First, the follow-up period was only 6 months or until discontinuation of treatment, so the long-term outcome of overall and progression-free survival could not be observed. Second, it is vital that clinicians log into the system and see the patients’ reports before every consultation. The ePRO app must be implemented in such a way that the process is embedded as part of routine care. In this regard, it is important that the ePRO app is easily accessible to medical staff (ie, integrated in the electronic health record) to be successful. Third, some patients’ adherence decreased over time, so the methods of improving adherence need to be considered in future research.

## Conclusions

To the best of our knowledge, this is the most up-to-date multicenter randomized clinical trial with a large sample to assess the efficiency of a follow-up model for patients receiving ICIs. Our findings show that the ePRO follow-up model helped to improve the safety and QOL of patients receiving immunotherapy and reduced follow-up time. This novel approach may provide reliable information and management recommendations.

## Notes

Supplement 1.
Trial Protocol

Supplement 2. **eMethods 1.** PRO App Information **eMethods 2.** Self-Reporting Form of irAEs

Supplement 3.
Data Sharing Statement
