# Supplementary material for: Efficiency of Electronic Health Record Assessment of Patient-Reported Outcomes After Cancer Immunotherapy: A Randomized Clinical Trial
Source: JAMA Netw Open. 2022 Mar 31;5(3):e224427. doi: 10.1001/jamanetworkopen.2022.4427 (PMC8972037; doi:10.1001/jamanetworkopen.2022.4427)
Supplement: Supplement 1. — Trial Protocol [file jamanetwopen-e224427-s001.pdf]

Efficiency of an Electronic Follow-up Model for Assessing Patient-Reported Outcomes after Cancer Immunotherapy: A Randomized Controlled Trial (Protocol)

Background

The oncology treatment landscape has been changed by immunotherapy, represented by immune checkpoint inhibitors（ICIs）. Monoclonal antibody therapy is mainly directed against cytotoxic T lymphocyte-associated antigen 4, CTLA4）programmed death-1 receptor（pd1）and its ligand1 (pdl1). The FDA has approved it to treat a variety of solid cancers and Hodgkin's lymphoma. A growing number of oncology patients may benefit from this, but at the same time, patients may experience immune-related adverse events（irAEs）. irAEs are non-specific responses produced by the immune system, affecting almost all tissues and organs. The most frequently involved sites are the skin, colon, endocrine system, liver, and lung, while there are few irAEs in the heart, liver, kidney, nerves, and eyes. Studies have shown that 90% of patients treated with single-agent ICI will experience varying degrees of irAEs, primarily mild to moderate. However, up to 43% of these patients apply ipilimumab, and about 20% of patients receiving PD1/PDL1 therapy will experience grade 3 or higher irAEs, such as severe enterocolitis, pneumonia, and myocarditis. Failure to identify and treat patients promptly can result in interruption of treatment in about 20% of patients and associated death in about 2% of patients.

Unlike chemotherapy-induced toxicity, irAEs are delayed reactions that typically occur months after administration or even withdrawal and last longer. The risk of irAEs is compounded by the fact that irAEs can be easily confused with other disease symptoms or overlooked, leading to delays in treatment. Medical staffs and patients should be vigilant for early identification of irAEs and timely intervention to ensure patient safety. The current strain on health care resources, the vast majority of time patients spend outside the hospital,

and the lack of supervision by health care workers further increase the risk of post-medication use. Effective and long-term out-of-hospital management of patients can play an essential role in the early detection and recognition of irAEs. If intervening on time can ensure the safety and efficacy of patients applying immunotherapy.

Foreign studies have shown that oncology patients with PRO follow-up systems can have a better prognosis than patients with regular outpatient follow-up, as evidenced by lower emergency department visits, a better quality of life, and more prolonged survival.

This system can be applied to monitor the status of mobile clients to detect serious treatment side effects at any time, to remind patients to go to the hospital on time. It can also provide doctors with the patient's indescribable discomfort symptoms during follow-up visits, improving the efficiency of the outpatient approach. Such systems can be safer for patients and reduce the occurrence of severe adverse reactions while also reducing the workload of health care workers. Given the emerging therapeutic approach of immunotherapy and the specificity of irAEs, exploring effective patient management to improve patient safety should be a priority for medical professionals; however, there is a lack of research findings in this area.

Objectives

A randomized controlled trail was designed to compare the outcomes between Patient-Reported Outcomes(PRO) and traditional follow up models in Cancer Immunotherapy. To explore the effects of ePROs mode on improving the safety including reducing the incidence of severe adverse reactions, unexpected emergency visits(ER), discontinue of treatment or death, improving quality of life and saving time of patients.

Methods

This study was an open label, multicenter randomized control trial comparing

the follow up outcomes among intervention group and control group among patients who treated with cancer immunotherapy . Randomization occurred immediately after a participant provided informed consent at each center. Participants were randomly assigned to the control group or intervention groups by computer system.

Patients

Enrollment started in September 2019, the study ended when the last patients were follow up by 6 month. Eligible individuals for the study were those who:1) were receiving cancer immunotherapy, 2) were aged ≥18 years, 3)Eastern cooperative oncology group (ECOG) performance status was 0 or 1, 4)life expectancy was at least 6 months,5) were also willing to complete the follow-up process according to the protocol, 6) could use smartphones or computers to include information in the App with or without the help of their caregivers were included in the study. Exclusion criteria: 1) diagnosed mental disease, 2)psychiatric symptoms that interfered with daily life and communication, 3) refused to participate in the study.

Sample size

Pass software was used to calculate the sample size. It was assumed that the incidence of severe adverse reactions in the test group was 10% lower than that in the control group. The unilateral Z test was applied; A =0.05, β=0.1. The results showed that there were 96 patients in each group; the data for 10% were determined to be lost during the follow-up process. The final sample size of each group was determined to include at least 103 cases considering the loss to follow up, the total sample size included 300 cases.,

Intervention

Follow up Team The ePRO App will be built and maintained by computer specialists from the Aistarfish Technology Co.,Ltd. A follow-up team that

included 1 oncology specialist and 2 nurses was conclude in every center. Every center will have a unique account of APP and the team were responsible for follow-up to their own patients. All researchers had at least 2 years of experience in ICIs therapy and received a training about research program, APP using and follow up process before study initial.

Control group

Researchers educated patients and their caregivers about immunotherapy and common symptoms of irAEs at the baseline. Patients was followed up by nurses as traditional model which including clinic visits every 21 days and via the telephone every 3 months. Patients can come to clinic when feel discomfort.

Intervention group

Patients in intervention group were assisted registered and followed up via the ePRO follow-up APP. The APP which contain questionnaire of common symptoms and image recognizen function of examination results to evaluating the occurrence and grades of typical irAEs according to the guidelines.
Patients complete questionnaire weekly and upload pictures of examination results between visits on their mobile or computer. The APP will sent a reminder message to patients who did not reply for two days delay. If there was still no reply, telephonic follow-up will be conducted.
The APP also had an algorithm for assessing severity of symptom or examining results according to NCI-CTCAE [18-21].When the grade 1/2 irAEs occurrence, the standardized advices were sent to relative patients by App automatically. If 3/4 irAEs were reported, the APP will alert health cares by sending alarms via text message, email, and APP at once. The health cares would immediately follow up via telephone and comprehensively evaluate the patient's situation. If serious AEs were still suspected, patients would be adviced to go to the emergency department, and evaluate and treat the patient

as soon as possible(See figure1).

Measures

Demographic characteristics and clinical conditions

Demographic information, including the age, sex, educational background, and marital status of patients, was recorded. Information regarding clinical conditions, including the primary cancer type, treatment, number of ER visits, and survival status, was also collected.

Relative indexes of IrAEs

Data regarding irAE incidence and grades, the rate of occurrence of grade 3/4 irAEs (cases/ total cases), and rate of treatment discontinuation and death owing to irAEs were collected.

Quality of Life

Quality of Life c-30 (QLQ c-30) was used to measure the QOL of subjects every 3 months. This tool, designed by Aaronson *et al.* [22], evaluates the ability of patients to adapt to life. It was translated into Chinese by Chonghua *et al*. [23], and the Chinese version was determined to be reliable. QLQ c-30 was comprised of 30 items, including 5 function dimensions (physical, role, emotional, perception, and social functions), 9 symptom dimensions (fatigue, nausea and vomiting, pain, shortness of breath, insomnia, loss of appetite, astriction, diarrhea, and economic difficulty), and 1 health situation. Two of the health situation items were assigned scores between 1 to 7, while the others were evaluated using the Likert 4 grade score. Based on the rules for assigning scores, we transformed every dimensional score to a standard score ranging from 0-100. The functional dimension score and the health situation score were positively correlated with patient QOL, while a high symptom dimension score was correlated with a severe manifestation.

Average time consuming

Researcher record the time consuming of each follow up. Average time consuming (total time /number of follow up per patient) of each follow up were compared between two groups.

Research route

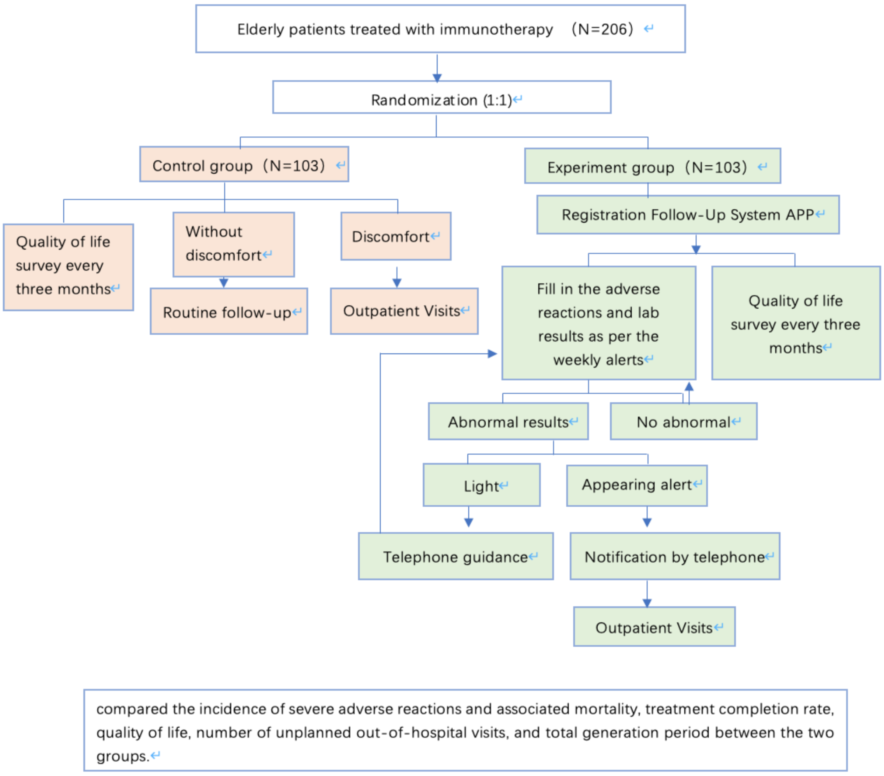

Expected solutions for critical problems and difficulties

Compared with the traditional follow-up model, whether the management model of the PRO follow-up system can reduce the incidence of severe irAEs and associated mortality after medication in immunotherapy patients. The application of the PRO follow-up system can improve patient safety by reducing the incidence of severe adverse reactions and associated mortality, the number of ER visits, treatment discontinue rate, quality of life, and overall

survival and time consuming in immunotherapy patients compared to the traditional follow-up model.

The difficulty of the study is how to improve the response rate of patients to the follow-up system. The solutions including firstly, the purpose and significance of the study are fully explained before the patients are enrolled to obtain their cooperation. Second, the system will send task reminders to the platform again for patients who do not answer after the timeout. If they still do not answer, there will be a telephone follow-up to ensure the integrity of the follow-up information.

Feasibility analysis

1) Theoretically feasible： This research on this topic is based on previous studies. The project is based on a sufficient basis, and the preliminary results are persuasive.

(2) Technically feasible: The group members include senior oncologists and nurses, and as the leader of the group, we have undertaken several related scientific research projects and treated nearly 1,000 patients receiving immunotherapy in recent years, so we have rich experience in the identification and management of related adverse reactions. As the principal investigator, we also conducted a randomized controlled study of the PRO follow-up system, with excellent results and a mature approach.

(3) Feasible conditions: Immunotherapy is currently one of the primary treatment modalities in our department, and more than 500 immunotherapy patients are now admitted annually. Hence, it is feasible to obtain study subjects.

Features and Innovations

Immunotherapy is currently one of the hottest directions in oncology

treatment, with an increasingly wide range of applications, and there is growing concern about how to ensure patient safety. However, domestic and international studies on patient management have been rarely reported. This study was conducted to manage patients on such new drugs to facilitate drug promotion and ensure patient safety and efficacy of drug administration. This study has more significant clinical implications for the management of patients on such new drugs in terms of drug promotion, ensuring safe patient access, and ensuring drug efficacy.

Foreign literature confirms that the PRO follow-up system leads to a better prognosis in oncology patients. However, there is a lack of studies reported in immunotherapy patients. In a context where domestic follow-up is still lacking, this study investigates the effectiveness of this new follow-up model, which can provide a basis for exploring effective follow-up models and has value for replication.

The present study is innovative, practical and generalized from theoretical and technical aspects.
