# Supplementary material for: Efficiency of Electronic Health Record Assessment of Patient-Reported Outcomes After Cancer Immunotherapy: A Randomized Clinical Trial
Source: JAMA Netw Open. 2022 Mar 31;5(3):e224427. doi: 10.1001/jamanetworkopen.2022.4427 (PMC8972037; doi:10.1001/jamanetworkopen.2022.4427)
Supplement: Supplement 2. — eMethods 1. PRO App Information eMethods 2. Self-Reporting Form of irAEs [file jamanetwopen-e224427-s002.pdf]

Supplementary Online Content

Zhang L, Zhang X, Shen L, Zhu D, Ma S, Cong L. Efficiency of electronic health record assessment of patient-reported outcomes after cancer immunotherapy: a randomized clinical trial. *JAMA Netw Open*. 2022;5(3):e224427. doi:10.1001/jamanetworkopen.2022.4427

eMethods 1. PRO App Information
eMethods 2. Self-Reporting Form of irAEs

This supplementary material has been provided by the authors to give readers additional information about their work.

© 2022 Zhang L et al. *JAMA Network Open*.

eMethods 1. PRO App Information

1.APP develop process:

We used kotlin language to develop Android App in the android studio compiler provided by Google， and we use Object-c language to develop iOS App in the Xcode compiler provided by Apple.

The technology called that collected the ePROs was Mass client information collection technology

2.Data collect and store:

We use IRAS(Intelligent real-time information acquisition system) to collect data, and store data in an independent and dedicated MySql database.

3. Information protect:

A) Data transmission security: TLS+Digital Signature technology

B) Data storage security: AES encryption algorithm

C) Data backup security: the master/slave database mechanism of MySql backs up data to the slave database in real time. Full data is backed up once a week and incremental data is backed up once a day

4. **The algorithm used to manage each potential irAE**: Masking algorithm and Decision tree Sort algorithm

© 2022 Zhang L et al. *JAMA Network Open*.

eMethods 2. Self-Reporting Form of irAEs

Dear patients, to ensure your safety during treatment, please note that if you have any of the following problems, please check appropriate box to report them, and we will deal with them promptly. (yellow parts are alert thresholds)

Part A. Do you experience any of the following discomforts? Please tick the number that matches your condition

1. Rash on the body, covering an area of the body surface (for example, face 4.5%, one arm 4.5%, one leg 9%)
1) Less than 10%
2) 10% to 30%
3) >30% or range 10%-30% with marked itching
4) >30% with discomfort such as erythema, large blisters, peeling epidermis

2.Abnormal bowel movements
1) None
2) Watery stools more often than before, but more often than <3 times per day
3) Watery stools 4-6 times more per day than before, or abdominal pain or bloody stools or nausea or nocturnal episodes
4) Watery stools >6 times a day more often than before, or episodes within 1 hour of a meal

3.Respiratory discomfort
1) None
2) No sensation, changes on chest radiograph or CT, suggesting pneumonia
3) Mild cough or dyspnoea or chest pain that does not interfere with sleep
4) Severe cough or dyspnoea or chest pain that interferes with sleep

4. Cardiac discomfort
1) None
2) palpitation
3) Pain in the precordial region
4) Chest tightness
5) Arrhythmia, feeling of a missed beat or fast and slow

5. Joint pain
1) None
2) Minor pain or swelling of the joint, not affecting movement
3) Severe pain that interferes with daily life
4) Unable to carry out daily activities

© 2022 Zhang L et al. *JAMA Network Open*.

6. Eye discomfort(Photophobia and tearing/ Redness of the eyes/ Blurred vision/ Dry, sore eyes)
1) None
2) Minor discomfort, not affecting daily life
3) Severe discomfort that interferes with daily life
4) Unable to carry out daily activities

7. Other symptoms (Mood or appetite change / Thirst, excessive urination / Headache)
1) None
2) Minor discomfort, not affecting daily life
3) Severe discomfort that interferes with daily life
4) Unable to carry out daily activities

Part B Please upload pictures of your laboratory results
1.Complete blood count
2.Comprehensive metabolic panel
3. Thyroid function

Following are standards of image recognition of laboratory results((yellow parts are alert thresholds)

1)Complete blood count
Ⅰ. Blood count
1. White blood cells
1) Not tested
2) 3000-4000 x10^9/L
3) 2000-3000 x10^9/L
4) 1000-2000 x10^9/L
5) <1000 x10^9/L

2. hemoglobin
1) Not tested
2) Below normal, but >95 g/L
3) 80-90 g/L
4) 65-80 g/L
5) <65 g/L

© 2022 Zhang L et al. *JAMA Network Open*.

3. Platelets
1) Not tested
2) 75-100 $x10^9$/L
3) 50-75 $x10^9$/L
4)25-50 $x10^9$/L
5)<25 $x10^9$/L

Comprehensive metabolic panel
1Liver function
1) Not tested
2) ALT or AST below 3 times normal or bilirubin between 1 and 1.5 times normal
3) ALT or AST between 3-5 times normal or bilirubin between 1.5-3 times normal
4) ALT or AST between 5-20 times normal or bilirubin between 3-10 times normal
(5) ALT or AST above 20 times normal or bilirubin >10 times normal

2 Renal function
1) Not tested
2) Elevated, at 1-1.5 times the upper limit of normal
3) >1.5-3 times upper limit of normal or
4) >3-6 times the upper limit of normal
5) >6 times the upper limit of normal or uric acid >10mg/dl

3.Blood glucose
1) Not tested
2) 6.1-8.9 mmol/L
3) 8.9-13.9 mmol/L
4) 13.9-27.8 mmol/L
5) 27.8 or more

4. Amylase
1) Not tested
2) 1-1.5 times normal
3) 1.5-2 times normal
4) 2-5 times normal
5) 5 times or more normal

5. Lipase
1) Not tested
2) 1-1.5 times normal
3) 1.5-2 times normal
4) 2-5 times normal
5) 5 times or more normal

© 2022 Zhang L et al. *JAMA Network Open*.

6. Cardiac enzymes :
1) Not tested
2) CK, CK-MB,cTNI higher than normal

Tyroid function
1）Nomal
2）Thyroid-stimulating hormone>10 or <0.01
3）Abnormal free thyroxine (FT4)

© 2022 Zhang L et al. *JAMA Network Open*.
