# Supplementary material for: Efficiency of Electronic Health Record Assessment of Patient-Reported Outcomes After Cancer Immunotherapy: A Randomized Clinical Trial
Source: JAMA Netw Open. 2022 Mar 31;5(3):e224427. doi: 10.1001/jamanetworkopen.2022.4427 (PMC8972037; doi:10.1001/jamanetworkopen.2022.4427)
Supplement: Supplement 3. — Data Sharing Statement [file jamanetwopen-e224427-s003.pdf]

Data Sharing Statement

Zhang. Efficiency of Electronic Health Record Assessment of Patient-Reported Outcomes After Cancer Immunotherapy. *JAMA Netw Open*. Published March 31, 2022. doi:10.1001/jamanetworkopen.2022.4427

Data
Data available: No

Additional Information
Explanation for why data not available: The datasets used and /or analyzed during the current study are available from the corresponding author on reasonable request.
